# Ceramide-Enriched Membrane Domains Contribute to Targeted and Nontargeted Effects of Radiation through Modulation of PI3K/AKT Signaling in HNSCC Cells

**DOI:** 10.3390/ijms21197200

**Published:** 2020-09-29

**Authors:** Riad Ladjohounlou, Safa Louati, Alexandra Lauret, Arnaud Gauthier, Dominique Ardail, Nicolas Magne, Gersende Alphonse, Claire Rodriguez-Lafrasse

**Affiliations:** 1 Laboratoire de Radiobiologie Cellulaire et Moléculaire, UMR CNRS 5822/IN2P3, IP2I, PRISME, Univ Lyon, Université Lyon 1, Faculté de Médecine Lyon-Sud, 69921 Oullins, France; safa.louati@icloire.fr (S.L.); alexandra.lauret@etu.univ-lyon1.fr (A.L.); arnaud.gauthier@univ-lyon1.fr (A.G.); dominique.ardail@univ-lyon1.fr (D.A.); nicolas.magne@icloire.fr (N.M.); gersande.alphonse@univ-lyon1.fr (G.A.); 2 Département de Radiothérapie, Institut de Cancérologie de la Loire—Lucien Neuwirth, 42270 Saint Priest en Jarez, France; 3 Hospices Civils de Lyon, Centre Hospitalier Lyon-Sud, 69495 Pierre-Bénite, France

**Keywords:** ceramide, lipid rafts, bystander effect, radioresistance, AKT, HNSCC

## Abstract

We investigated the potential involvement of ceramide-enriched membrane domains in radiation-induced targeted and nontargeted effects using head and neck squamous cell carcinoma with opposite radiosensitivities. In radiosensitive SCC61 cells, the proportion of targeted effects was 34% and nontargeted effects killed 32% of cells. In contrast, only targeted effects (30%) are involved in the overall death of radioresistant SQ20B cells. We then demonstrated in SCC61 cells that nontargeted cell response was driven by the formation of the radiation-induced ceramide-enriched domain. By contrast, the existence of these platforms in SQ20B cells confers a permissive region for phosphatidylinositol-3-kinase (PI3K)/AKT activation. The disruption of lipid raft results in strong inhibition of PI3K/AKT signaling, leading to radiosensitization and apparition of nontargeted effects. These results suggest that ceramide-enriched platforms play a significant role in targeted and nontargeted effects during radiotherapy and that drugs modulating cholesterol levels may be a good alternative for improving radiotherapy effectiveness.

## 1. Introduction

Accumulated evidence shows that the biological effects of ionizing radiation can be expressed in cells that are not directly hit by particles but are in contact with or close vicinity to irradiated cells [1,2,3]. This nontargeted effect of radiation, also called bystander effect, has been mainly observed after low doses and is induced by low or high linear energy transfer (LET) radiation [4,5]. Nontargeted effects are associated with a lack of dose–effect relationships [6,7]. The modifications of biological functions include cell death [8], DNA damage [9], apoptosis [10], genomic instability [11], and radio-adaptive responses [12]. Two major mechanisms mediate the bystander response: (i) the transmission of signaling molecules through gap junctions when cells are in contact [13,14] and (ii) the release of soluble factors by irradiated cells in the extracellular environment [8,15,16]. The precise nature of factors that mediate the nontargeted effect is unknown, but reactive oxygen and nitrogen species (ROS/RNS) and various cytokines have been involved [17,18,19]. Other studies [20,21,22] have also demonstrated the involvement of plasma membrane signaling in the bystander effect via the generation of ceramide (considered as a mediator of radiation), leading to activation of the mitogen-activated protein kinases (MAPK) and other pathways, which then transduce amplified signals into the nucleus.

Ceramide is the most well-studied sphingolipids in cancer due to its role in cellular differentiation and induction of death. It is generated in a plasma membrane upon hydrolysis of sphingomyelin (SM) by acid or neutral sphingomyelinase (A or NSMase). Ceramide is also synthesized either by de novo synthesis occurring in the endoplasmic reticulum or from reacylation of the sphingosine in the salvage pathway [23]. At the cell membrane, ceramide molecules interact with each other, resulting in the formation of stable and tightly packed ceramide-enriched membrane microdomains. These microdomains spontaneously associate to form large ceramide-enriched membrane macrodomains or platforms, also called lipid rafts [24,25]. Lipid rafts are small (10–200 nm), heterogeneous, highly dynamic, cholesterol- and sphingolipid-enriched nanodomains that have the potential to fuse and form microscopic domains (>300 nm) through protein–protein and protein–lipid interactions. These domains are present in both the inner and the outer leaflets of the asymmetric cell membrane and have been involved in the compartmentalization of different cellular processes, including intracellular trafficking, membrane signaling [26,27], and cellular functions such as regulation of apoptosis and cell proliferation [28,29,30].

Several studies have shown that these membrane domains contain high concentrations of different receptors, like glycosylphosphatidylinositol (GPI)-anchored hormone receptors, inositol-1,4,5-trisphosphate receptors, epidermal growth factor receptors (EGFR), or platelet-derived growth factor receptors. Other molecules enriched in lipid rafts and involved in cell signaling have also been reported such as protein kinase C, phospholipase C, SRC family kinases, heterotrimeric G protein subunits, nonreceptor tyrosine kinases, or phosphatidylinositol-3-kinase (PI3K) [31,32].

Protein kinase B (PKB) also known as AKT, a serine/threonine kinase, is a central mediator of cell survival, and its deregulation has been involved in cancer progression [33,34]. PI3K is necessary for the activation of AKT. Indeed, upon receptor-mediated activation, PI3K phosphorylates phosphatidylinositol-4,5-bisphosphate (PIP_2_), thus generating phosphatidylinositol-3,4,5-triphosphate (PIP_3_). PIP_3_ recognizes specifically a pleckstrin homology (PH) domain of AKT, thus triggering activation by translocation from the cytosol to the plasma membrane. PIP_3_ also binds phosphoinositide-dependent kinase 1 (PDK1), leading to activation of AKT [35]. Several studies have reported that both AKT [36,37] and PDK1 [38,39] are localized into lipid rafts, suggesting the possible involvement of ceramide-enriched domains in PI3K/AKT pathway activation.

Previous data from our laboratory [40] showed that ceramide-enriched membrane can play a key role in the response to irradiation of two human head and neck squamous cell carcinoma (HNSCC) incidences of opposite radiosensitivities. However, the implication of nontargeted effect was never studied.

Therefore, this study is designed to investigate the role of lipid raft in targeted and nontargeted effects of irradiation in HNSCC cells to improve our knowledge about the mechanisms regulating their radiosensitivity. We found that, in addition to the direct effects of radiation, nontargeted effects were significant in radiosensitive SCC61 cells, involving lipid raft formation that could lead to cell death. However, the preexistence of these platforms in radioresistant cells led to an insensitivity of these cells to nontargeted effects. These results suggest that, depending on its genesis and/or intrinsic nature of cells, ceramide-enriched domains may modulate both cell survival and/or death. The use of cholesterol-depleting drugs should therefore be experienced in the future to improve treatment of radioresistant HNSCC.

## 2. Results

### 2.1. Nontargeted Effects Were Observed in SCC61 Cells But Not in SQ20B Cells

**Figure 1:**
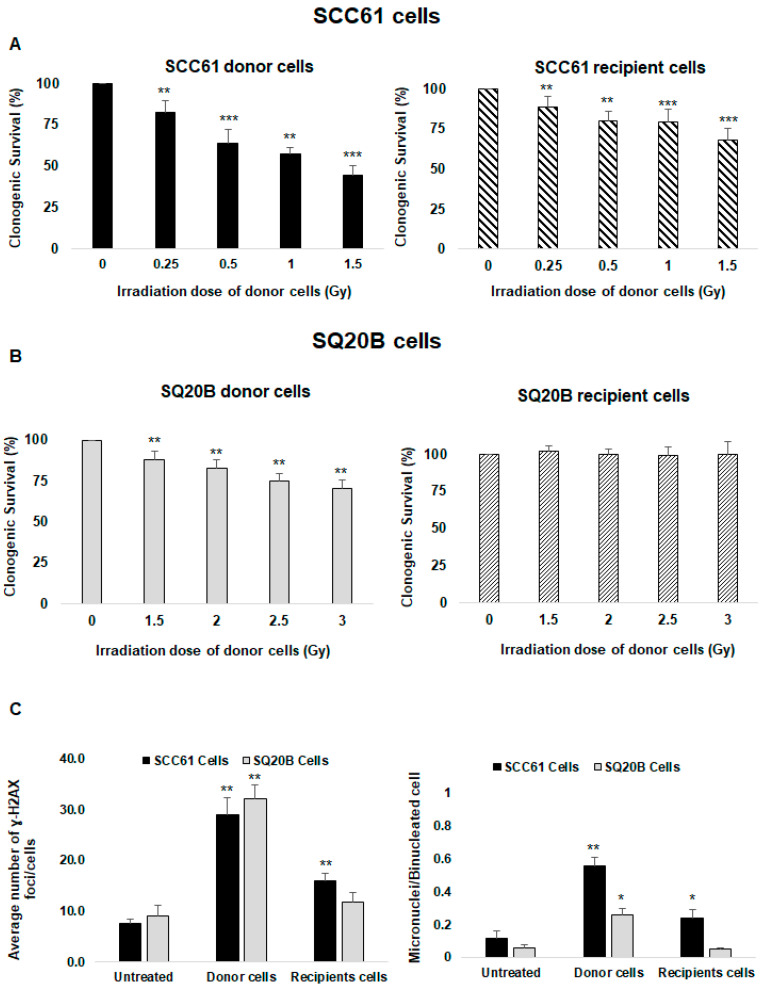
Assessment of targeted and nontargeted effects: clonogenic survival assays were performed in donor and recipient cells 12 days following X-ray irradiation (donor cells) or incubation in culture medium in which donor cells had been cultured for 2 h (recipient cells) as described in the Materials and Methods section. Survival was measured in (**A**) SCC61 cells and (**B**) SQ20B cells. (**C**) The average numbers of H2A.X foci and micronuclei per cell were determined using immunofluorescence assay in SCC61 and SQ20B donor cells (1.5 Gy and 3 Gy, respectively) and in corresponding recipient cells. The results are the mean ± SD of three experiments performed in triplicate. * *p* < 0.05, ** *p* < 0.01, and *** *p* < 0.001 compared with untreated cells.

The cell survival of SCC61 donor cells was strongly reduced in response to irradiation; at the highest dose (1.5 Gy), it decreased to 44.8% ± 5.4% (Figure 1A left panel). The plating efficiency (PE) for unirradiated cells was 38%. When the conditioned medium (CM) was transferred from donor cells to recipient cells, a strong cytotoxicity was also observed. The survival was reduced to 68.0% ± 7.6%, indicating the existence of nontargeted effects in SCC61 cells (Figure 1A right panel).

Consistent with our previous study [41], SQ20B cells were more radioresistant than SCC61 cells. The cell survival decreased to 88.1% ± 5.3% at the 1.5 Gy dose and to 70.5% ± 5.2% after 3 Gy (Figure 1B left panel). No cell killing was observed in SQ20B recipient cells when incubating with the CM of SQ20B donor cells (Figure 1B right panel). Because the nontargeted cytotoxicity was the highest at 1.5 Gy in radiosensitive SCC61 cells, further experiments were performed at this dose whereas a 3 Gy dose was chosen for the radioresistant SQ20B cells.

### 2.2. DNA Damage Confirms the Presence of Nontargeted Effects Only in Radiosensitive SCC61 Cells 

Next, we quantified the number of γ-H2A.X foci as a reflection of DNA double-strand breaks (DNA DSBs). The mean numbers of γ-H2A.X foci per cell were 28.9 ± 3.5 in SCC61 donor cells at 30 min postirradiation, 16 ± 1.4 in recipient cells, and 7.6 ± 0.8 in nontreated cells (Figure 1C left panel and Supplementary Figure S1A), confirming the occurrence of nontargeted effects in SCC61 cells. As misrepaired or unrepaired DNA DSBs can lead to chromosomal aberrations, we also measured the formation of micronuclei. The number of micronuclei was significantly increased in SCC61 donor cells (0.56 ± 0.05) and their corresponding recipient cells (0.24 ± 0.05), while in the untreated cells, the level was 0.12 ± 0.04 (Figure 1C right panel and Figure S1B).

For radioresistant SQ20B cells, the quantification of the mean number of γ-H2A.X foci per cell at the 3 Gy dose was 32.1 ± 2.9 in donor cells, 11.0 ± 1.7 in the recipient cells, and 9.0 ± 1.9 in the untreated cells (Figure 1C left panel and Figure S1A). These data confirm that SQ20B cells are more resistant to radiation than SCC61 cells; also, the absence of DNA damage in corresponding recipient cells confirms that these cells are unable to respond to nontargeted effects. Compared with untreated cells, the number of micronuclei was increased in SQ20B donor cells while no cytotoxic effects were detected in SQ20B recipient cells (Figure 1C right panel and Figure S1B).

### 2.3. SQ20B Cells Are Able to Produce a Bystander Signal

**Figure 2:**
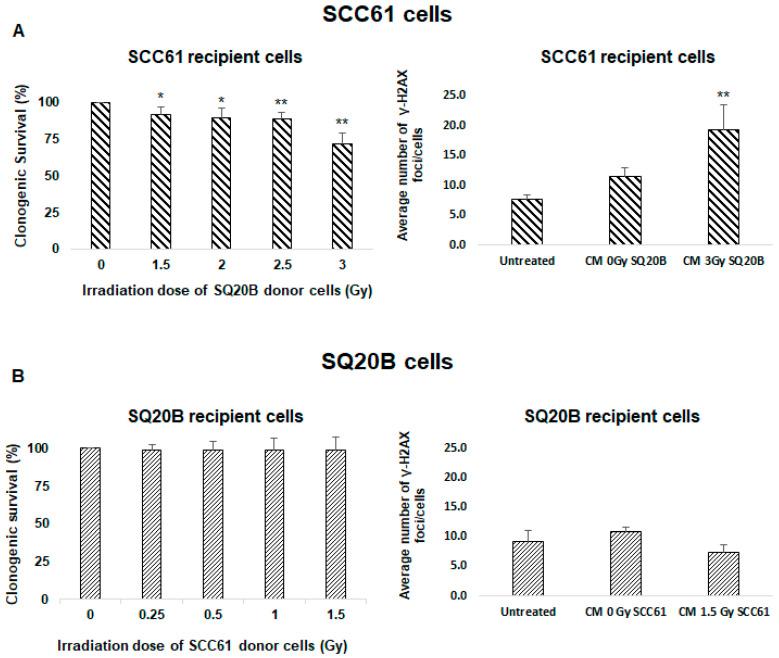
Bystander effectors are radio-induced in SQ20B cells. To assess whether SQ20B cells could induce bystander factors, culture mediums were exchanged between SCC61 and SQ20B cells and clonogenic survival and induction of γ-H2A.X foci were evaluated. (**A**) SCC61 recipient cells were incubated with conditioned medium from SQ20B donor cells, and (**B**) SQ20B recipient cells were cultured in medium from SCC61 donor cells. The results are the mean ± SD of three experiments performed in triplicate. * *p* < 0.05, ** *p* < 0.01, compared with untreated cells.

To understand the origin of SQ20B cell resistance to nontargeted effects, we first investigated whether the CM of SQ20B donor cells contains bystander factors that can induce this type of signaling. Therefore, SCC61 recipient cells were incubated with the CM from SQ20B donor cells and clonogenic survival was evaluated. We observed that survival was reduced to 71.3% ± 7.5% (Figure 2A left panel). These data suggest that, following a 3 Gy dose, the CM from SQ20B cells induces a nontargeted effect in SCC61 cells. This cytotoxicity was confirmed by the detection of the γ-H2A.X foci in SCC61 recipient cells treated with the CM from SQ20B donor cells (Figure 2A right panel). When SQ20B recipient cells were treated with the CM from SCC61 donor cells, no cytotoxicity occurred, as shown in Figure 2B. These findings suggest that SQ20B cells can induce bystander stimulations but not develop nontargeted responses originating from their own supernatant or from the CM of SCC61 radiosensitive cells.

### 2.4. Cell Membrane Reorganization Was Radio-Induced in Radiosensitive SCC61 Cells But Not in SQ20B Cells

**Figure 3:**
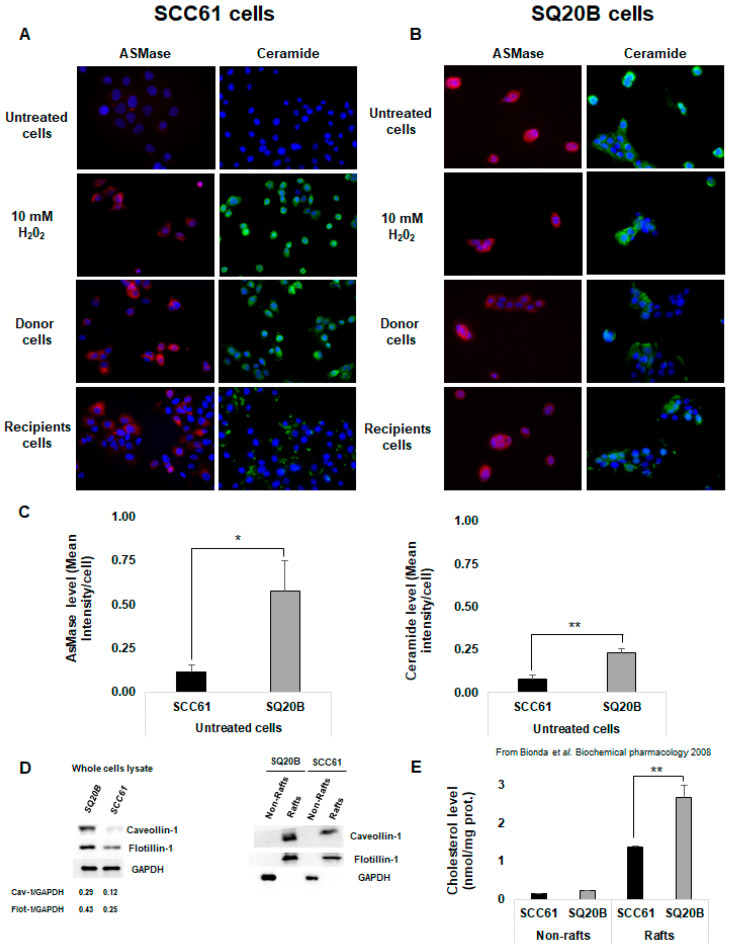
Cell membrane reorganization was radio-induced in radiosensitive SCC61 cells but not in radioresistant SQ20B cells. ASMase activation and ceramide production were measured using immunofluorescence in (**A**) SCC61 cells (donor and recipients) and (**B**) SQ20B cells (donor and recipients). (**C**) ASMase and ceramide levels in basal condition (untreated) in SCC61 and SQ20B cells were quantified using immunofluorescence images. (**D**) Lipid raft markers were assessed using western blotting in both SCC61 and SQ20B cells in the total lysate or in the fractionated Triton-soluble membrane (non-rafts) and lipid raft (rafts) fractions as described. (**E**) Cholesterol level was measured in non-raft and raft fractions isolated from SCC61 and SQ20B cells in basal conditions. The results are the mean ± SD of three experiments performed in triplicate. * *p* < 0.05, ** *p* < 0.01.

Because increasing evidence indicated that ceramide-enriched microdomains contribute to the bystander induction, we investigated whether radiation could affect the cell membrane organization of SCC61 and SQ20B donor cells. As previously reported [42], we found that, in the radiosensitive SCC61 cells, irradiation leads to ASMase activation, leading to ceramide generation (Figure 3A left panel). A similar trend was observed in SCC61 recipient cells. In contrast, neither ASMase generation nor radio-induced ceramide production were observed in SQ20B cells (Figure 3B right panel). However, immunofluorescence image analyses showed that, compared with SCC61 cells, in basal conditions, radioresistant SQ20B cells present a higher level of ASMase activity (×4.9; *p* = 0.028; Figure 3C left panel and Table S1) and ceramide concentration (×2.9; *p* = 0.008; Figure 3C right panel and Table S1). Because ceramide is known to cluster and form ceramide-enriched macrodomains, these results suggest the presence of lipid rafts in SQ20B cell membranes in the absence of irradiation.

To test this hypothesis, both untreated SCC61 and SQ20B cells were fractionated into raft and non-rafts domains and the membrane composition of cells in lipid raft markers was analyzed using western blot. Figure 3D shows that the SQ20B cell membrane is enriched in lipid raft protein markers such as caveolin-1 and flotillin-1 compared with SCC61 cells. This result is in agreement with our previous study [40], which demonstrated that the cholesterol level was twice as high (*p* < 0.01) in the SQ20B raft domain than in SCC61 cells (Figure 3E).

### 2.5. Lipid Rafts Are Involved in the Targeted and Nontargeted Responses in Radiosensitive SCC61 Cells

**Figure 4:**
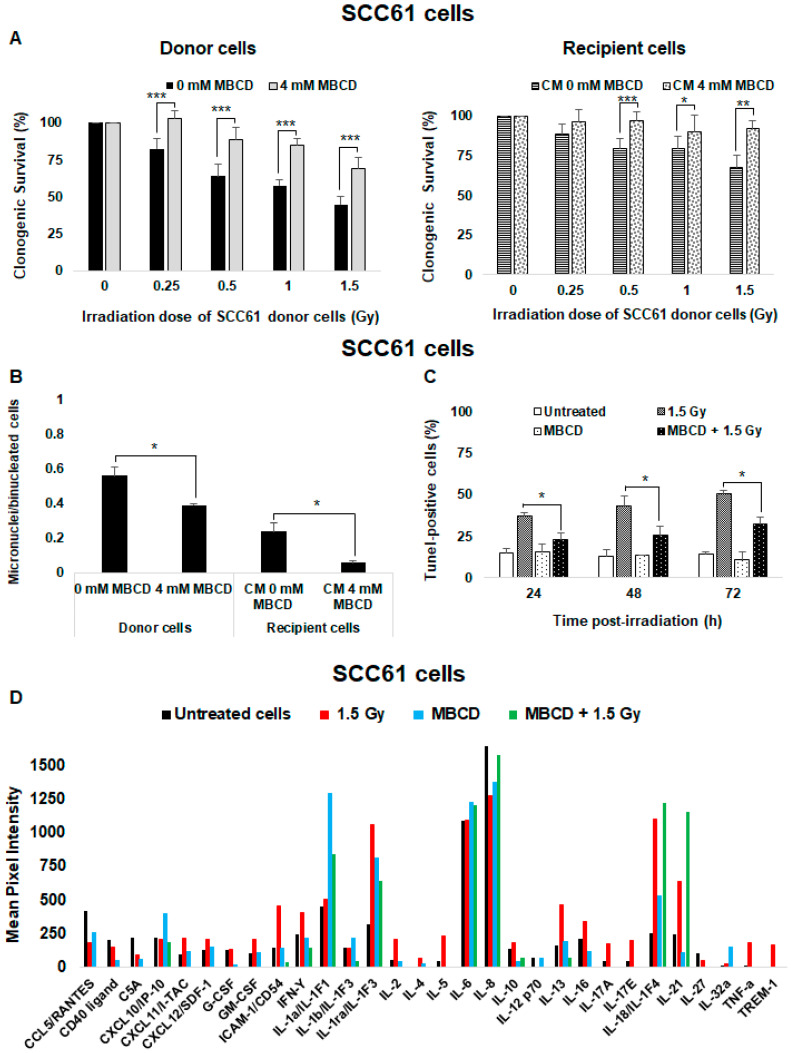
Role of lipid raft formation in radiosensitive SCC61 cells: SCC61 donor cells were preincubated for 30 min with 4 mM methyl-β-cyclodextrin (MBCD; lipid raft disruptor) before irradiation in the presence of MBCD. After irradiation, the medium was removed and (**A**) clonogenic survival was determined in donor cells and in recipient cells. (**B**) The number of micronuclei per cell was determined in donor cells in the presence or absence of MBCD and in their corresponding recipient cells. (**C**) Apoptosis was measured by a TUNEL assay in SCC61 donor cells preincubated or not with 4 mM MBCD for 30 min before irradiation. The results are the mean ± SD of three experiments performed in triplicate. * *p* < 0.05, ** *p* < 0.01, and *** *p* < 0.001 compared with cells treated only with radiation. (**D**) The expression of cytokines secreted in conditioned medium from untreated, irradiated (1.5 Gy), and MBCD + irradiated SCC61 cells was evaluated using the Human Cytokine Array (Proteome Profiler Array; R&D Systems, Minneapolis, MN, USA). For each cytokine, ImageJ software was used to determine pixel intensity.

To study the role of radiation-induced lipid rafts in radiosensitive SCC61 cells, cells were incubated with methyl-β-cyclodextrin (MBCD), a lipid raft disruptor which depletes cholesterol from the cell membrane. Upon irradiation, the clonogenic survival of both donor and recipient cells increased in the presence of MBCD (Figure 4A) from 44.8% ± 5.4% to 68.9% ± 7.5% in SCC61 donor cells and from 68.0% ± 7.6% to 91.9% ± 5% in their corresponding recipient cells. We also found that the micronuclei formation was lower in donor and recipient SCC61 cells after cholesterol depletion (Figure 4B). Furthermore, the percentage of apoptotic cells was significantly reduced (*p* < 0.05) following lipid rafts disruption in comparison to cells irradiated alone (Figure 4C).

Because lipid rafts are known to participate in signal transduction, we also analyzed the soluble factors released by SCC61 donor cells following the generation of cell membrane ceramide using human cytokine array (Figure 4D). Compared with CM from untreated cells, several cytokines were produced after irradiation. Pro-inflammatory cytokines such as interferon γ (IFN-Y) and TNF-α were considerably increased. ICAM-1 (InterCellular Adhesion Molecule); TREM-1 (Triggering receptor expressed on myeloid cells 1); and many interleukins such as IL-1ra, IL-2, IL-13, IL-17A, IL17-E, IL-18, and IL-21 were also increased following irradiation. However, after incubation with MBCD followed by irradiation, these cytokines were strongly reduced in CM from SCC61 donor cells, except IL-21 compared with untreated cells or cells only treated with MBCD, thus suggesting that radiation-induced lipid raft formation participates in nontargeted effects.

### 2.6. Cholesterol Depletion Sensitizes SQ20B Cells to Radiation and Induces the Nontargeted Response

**Figure 5:**
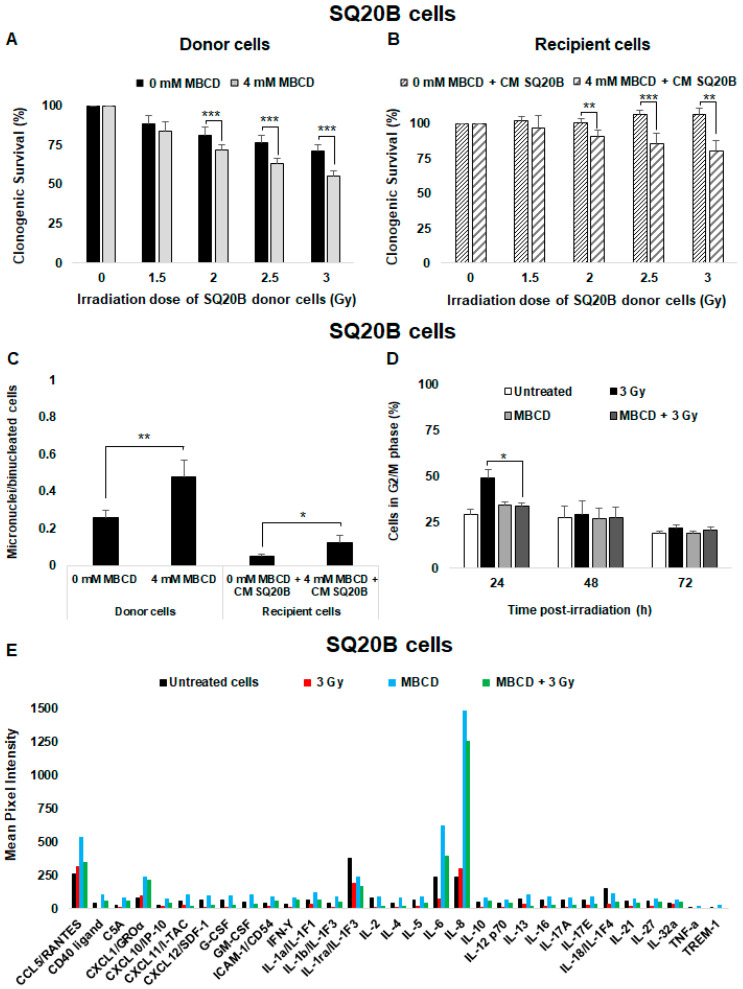
Lipid raft disruption sensitizes SQ20B donor cells to radiation and induces nontargeted effects. SQ20B donor cells were preincubated for 60 min with 4 mM MBCD (lipid raft disruptor) before irradiation in the presence of MBCD. After irradiation, the medium was removed and (**A**) clonogenic survival was determined in donor cells. (**B**) Clonogenic survival of SQ20B recipient cells was co-incubated or not with MBCD before transfer of the CM from SQ20B donor cells. (**C**) The number of micronuclei per cell in SQ20B donor and recipient cells in the presence or absence of MBCD was quantified. (**D**) The percentage of cells in G2/M phase was determined using flow cytometry in SQ20B donor cells in the presence or absence of MBCD. The results are the mean ± SD of three experiments performed in triplicate. * *p* < 0.05, ** *p* < 0.01, and *** *p* < 0.001 compared with cells treated only with radiation. (**E**) The expression of cytokines secreted in the CM from untreated, irradiated (3 Gy), and MBCD + irradiated SQ20B cells was evaluated using the Human Cytokine Array (Proteome Profiler Array; R&D Systems, Minneapolis, MN, USA). For each cytokine, ImageJ software was used to determine pixel intensity.

When SQ20B donor cells were preincubated with MBCD before irradiation, a significant reduction of clonogenic survival was observed compared with nontreated cells after low doses (Figure 5A) or high doses of irradiation (Figure S2A). Cell survival was reduced from 71.52 ± 3.7% to 55.20 ± 3.7% (*p* < 0.001) at 3 Gy. The survival of SQ20B recipient cells was similarly reduced in the presence of CM from SQ20B donor cells (Figure 5B) or CM from SCC61 donor cells (Figure S2B) after cholesterol depletion treatment. Moreover, incubation of SQ20B donor cells with MBCD before irradiation resulted in an increase in the number of micronuclei per cell (×1.8; *p* = 0.009; Figure 5C). This number in SQ20B recipient cells pretreated with MBCD was also higher than with cells incubated only with CM from SQ20B cells (Figure 5C). In agreement with our previous studies [41,42], cell cycle analyses showed a transient increase in the percentage of SQ20B cells in the G2/M phase at 24 h postirradiation compared with untreated cells (×1.7; *p* = 0.009; Figure 5D). This effect was not observed when SQ20B cells were incubated with MBCD before irradiation. In contrast, no G2/M phase blockade was observed in the absence or presence of MBCD in radiosensitive SCC61 cells (Figure S2C). However, no apoptosis occurred after exposure of SQ20B cells to radiation, even after pretreatment with MBCD (Figure S2D).

Next, CM from radioresistant SQ20B donor cells was analyzed using the human cytokine array (Figure 5E). Exposure to radiation did not change the cytokine secretion profile of SQ20B donor cells. Nevertheless, after lipid raft disruption with MBCD, the concentration of some pro-inflammatory cytokines such as IL-6 and IL-8 were significantly increased compared with irradiated or untreated cells. Interestingly, the combination of MBCD + irradiation did not induce a significant release of cytokines in SQ20B donor cells.

These results indicate that the presence of lipid rafts in the SQ20B cell membrane contributes to resistance to irradiation and the lack of radiation-induced nontargeted effects.

### 2.7. Lipid Raft Disruption Is Accompanied by Loss of AKT Phosphorylation in SQ20B Cells

**Figure 6:**
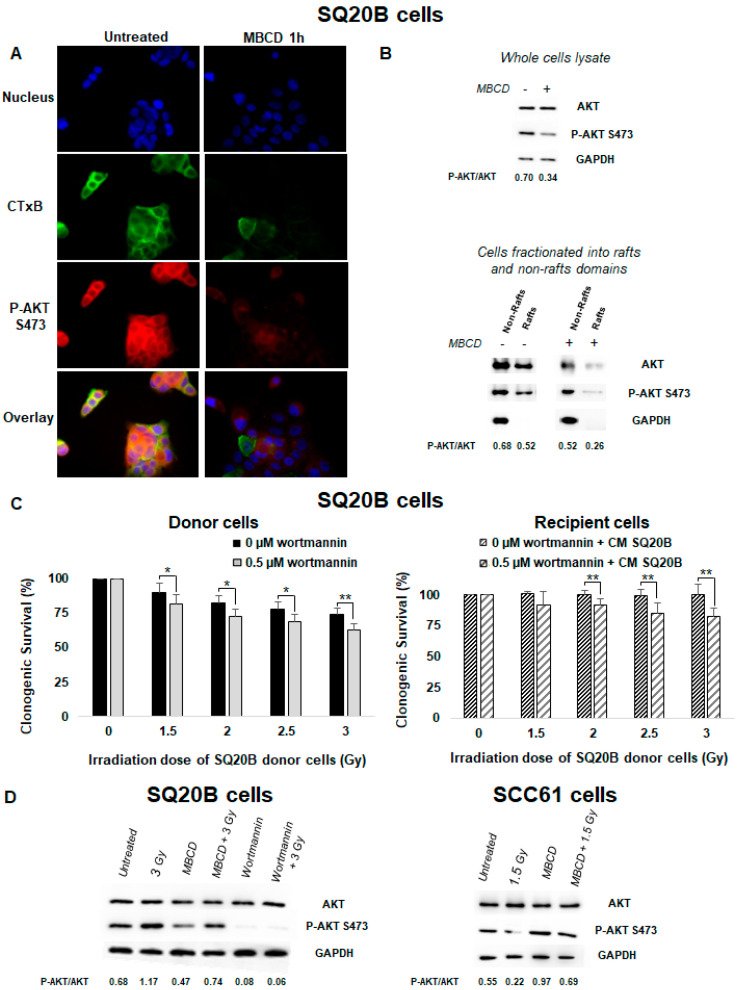
An endogenous AKT protein resides in lipid raft fractions of SQ20B cells and confers resistance to targeted and nontargeted effects. (**A**) SQ20B cells treated without (untreated) or with MBCD were incubated with Alexa-488-conjugated cholera toxin B (CTxB) following incubation with p-AKT S473. (**B**) SQ20B cells were pretreated in the presence or absence of MBCD, and total cell lysates (upper panel) or fractions isolated from non-raft and raft domains (bottom panel) were subjected to SDS-PAGE followed by western blotting for p-AKT S473. (**C**) SQ20B cells were preincubated or not with the phosphatidylinositol-3-kinase (PI3K) inhibitor (0.5 µM wortmannin) for 60 min followed by irradiation (donor cells) or CM from SQ20B donor cells (recipient cells), and clonogenic survival was determined. The results are the mean ± SD of three experiments performed in triplicate. * *p* < 0.05, ** *p* < 0.01, compared with cells treated only with radiation. (**D**) p-AKT S473 was expressed in total cell lysates of SQ20B and SCC61 cells in the presence or absence of MBCD or wortmannin.

To understand how lipid rafts can disrupt bystander signaling and can mediate the pro-survival effect in SQ20B cells, the signaling pathways modified by cholesterol depletion treatment were investigated. Several studies reported that AKT protein kinase activity is overexpressed in HNSCC cells [43,44]. Using immunofluorescence detection, we evaluated under basal conditions the presence of lipid rafts and the expression of AKT phosphorylation in SQ20B cells. Figure 6A shows that AKT co-localized with lipid raft, as visualized by cholera toxin B (CTxB) staining. Following lipid raft disruption, a strong inhibition of AKT phosphorylation was observed mainly in the raft domains (Figure 6B). These results confirm the existence of AKT, located within lipid raft domains of SQ20B cells.

### 2.8. PI3K/AKT Signaling Was Involved in the Absence of Nontargeted Response in Radioresistant SQ20B Cells

The PI3K/AKT signaling pathway is involved in cell growth and proliferation and is often dysregulated in many cancers. Using wortmannin, a specific inhibitor of PI3K, we investigated the potential role of the PI3K/AKT pathway in the absence of nontargeted response and resistance to radiation in SQ20B cells. As shown in Figure 6C (left panel and Figure S3A), pretreatment of SQ20B donor cells with wortmannin resulted in increased cell death compared with irradiation alone. SQ20B recipient cells showed also a reduction of their survival upon pretreatment with this PI3K inhibitor (Figure 6C right panel). After MBCD treatment, co-incubation of both SQ20B donor and recipient cells with wortmannin was followed by an increase in micronuclei formation (Figure S3B) and suppression of the G2/M phase arrest at 24 h postirradiation (Figure S3C). Moreover, the use of wortmannin before irradiation did not trigger apoptotic death in SQ20B cells (Figure S3D).

We also observed that irradiation alone did not change the expression of AKT phosphorylation in SQ20B cells compare with radiosensitive SCC61 cells (Figure 6D). However, inhibition of p-AKT was evidenced when SQ20B cells were pretreated with MBCD or wortmannin before irradiation. By contrast, when SCC61 cells were depleted of their cholesterol content, inhibition of p-AKT following radiation was eliminated. In addition, analysis of the secreted cytokines after inhibition of the PI3K/AKT pathway showed no change either in the CM of irradiated SQ20B cells or in the control (Figure S3E).

### 2.9. Relative Contribution of Targeted and Nontargeted Cytotoxicity

**Figure 7:**
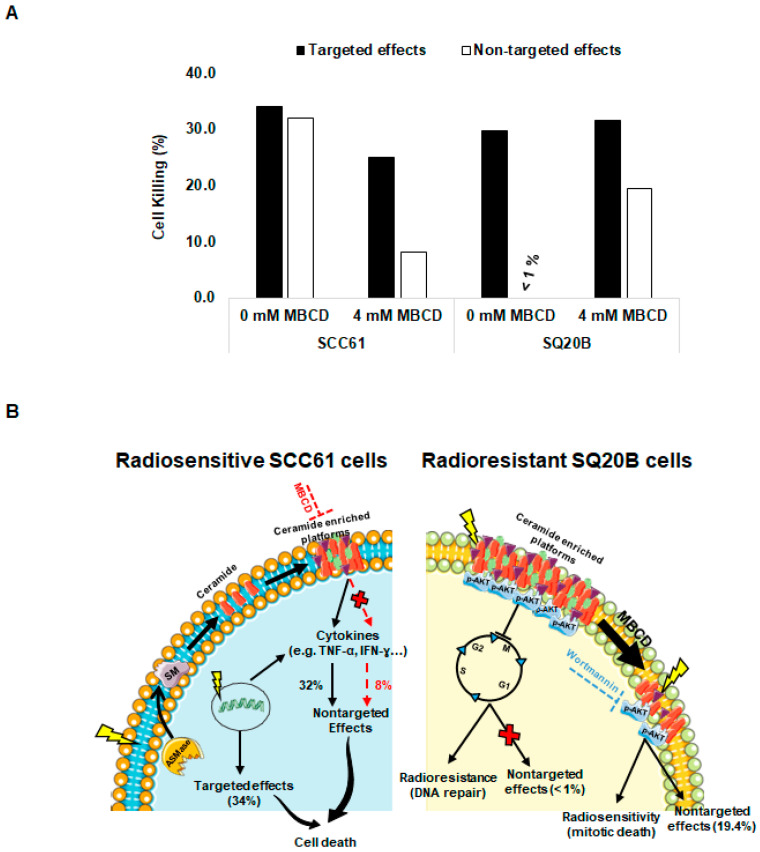
Contribution of targeted and nontargeted effects of radiation in radiosensitive SCC61 and radioresistant SQ20B cells: (**A**) the percentage of targeted and nontargeted effects to cell killing related or not to lipid raft disruption was determined using a Bliss independence mathematical model as described. (**B**) In radiosensitive SCC61 cells, irradiation led to targeted effects via the production of DNA damage or via activation of ASMase, which induces sphingomyelin hydrolysis to ceramide and phosphorylcholine and participates in the formation of ceramide-enriched platforms. These platforms can promote different signaling pathways including cytokine release, leading to nontargeted cytotoxicity. In the presence of a lipid raft disruptor (MBCD), these nontargeted effects were strongly reduced. Conversely, in radioresistant SQ20B cells, the preexistence of ceramide-enriched domains provides a suitable environment for AKT activation and contributes to resistance to radiation and the absence of nontargeted effects by promoting G2/M cell cycle arrest, allowing DNA repair. The disruption of lipid rafts produces AKT dephosphorylation and suppresses arrest in the G2/M phase cell cycle, leading to cell death and apparition of nontargeted effects. Similarly, inhibition of AKT survival pathways by wortmannin showed an increase of cell death and bystander response.

Using experimental data obtained from donor and recipient cells (Figure 1A,B), we could establish the relative contribution of targeted and nontargeted cytotoxicity in our two cell lines (Figure 7A). In radiosensitive SCC61 cells, targeted and nontargeted cytotoxic effects induced 34.1% and 32% of cell death, respectively (Figure 7A). This suggests that at least 50% of cell killing is due to nontargeted effects in SCC61 cells. By contrast, cell death resulted only from targeted effects (29.7%) in radioresistant SQ20B cells. Similarly, we determined the lipid raft-mediated targeted and nontargeted cytotoxicity. We showed that the contribution of lipid raft in the targeted cytotoxic effects represents about 27% (i.e., 9.1% of 34.1%) and about 75% for the nontargeted effects (i.e., 24% of 32%) in SCC61 cells. For SQ20B cells, lipid raft-mediated targeted effects were lower: 6% (i.e., 1.8% of 31.5%) and higher (98%) for nontargeted effects (19.2% of 19.4%). These data suggest that, after cholesterol depletion, the occurrence of nontargeted cytotoxic effect was mainly responsible for the radiosensitization observed in SQ20B cells. 

## 3. Discussion

In the present study, we show, on the one hand, that nontargeted effects via cell membrane signaling play a significant role in the response to irradiation and, on the other hand, that lipid raft disruptors such as MBCD could modulate the outcome of radiation.

Nontargeted, mediated cytotoxicity was evaluated using a media transfer protocol. We demonstrated a strong bystander response in the radiosensitive SCC61 recipient cells, whereas no toxicity was observed in SQ20B recipient cells. Our results also showed that, even though SQ20B cells could produce bystander factors, they were not affected by the presence of these radio-induced molecules. The cell membrane is one of the first targets for ionizing radiation. Indeed, several studies [23,45] established that, upon irradiation of plasma membrane, hydrolysis of the SM occurred via ASMase activation, generating ceramide and phosphorylcholine. The radio-induced ceramide can then spontaneously self-associate to form ceramide-enriched membrane platforms called lipid rafts which are capable of triggering many signaling pathways. Our results indicate that a similar mechanism seems to occur in radiosensitive SCC61 cells. We showed that irradiation induced an externalization of ASMase and its activation followed by ceramide generation in SCC61 donor cells. A similar trend was also observed in recipient cells, thus indicating the involvement of this event in nontargeted effects. Conversely, we found that radioresistant SQ20B cells spontaneously exhibited a high ASMase activity at the plasma membrane and thus a high level of membrane ceramide compared with the radiosensitive SCC61 cells in basal conditions (Figure 3C,D). Furthermore, the high cholesterol (a major component of lipid raft) content together with the abundance of lipid raft markers such as caveolin and flotillin suggest the existence of endogenous lipid raft platforms in the SQ20B cell membrane. However, no significant change was observed in the cell membrane organization of SQ20B donor cells after irradiation.

The contribution of lipid rafts in targeted and nontargeted effects was then investigated. When SCC61 cells were treated with MBCD before irradiation, we showed a significant decrease in cell death in both donor and recipient cells. Moreover, the production of bystander molecules such as TNF-α and IFN-Y [46] was eliminated after treatment with MBCD, thus resulting in a reduction of apoptotic cells death (Figure 4B right panel), which suggests a radioprotective effect of MBCD in radiosensitive SCC61 cells. By contrast, the disruption of lipid rafts in SQ20B cells led to increased cell death together with a bystander-like response. This was evidenced by the persistence of DNA damage (micronuclei) resulting from inhibition of G2/M cell cycle phase arrest after pretreatment of radioresistant SQ20B cells with MBCD followed by radiation. Similar results have been previously reported [47,48,49], which demonstrated the role of cholesterol-depleting agents in DNA repair after irradiation. However, our study shows that apoptosis does not play a major role in the cytotoxicity induced by lipid raft disruption in radioresistant SQ20B cells. Our results also demonstrated that no cytokines were released by irradiated SQ20B cells, thus suggesting that cytokines do not participate in radiation-induced nontargeted effects observed in these cells following lipid raft disruption. In addition to cytokines, many other molecules [50,51,52] secreted by irradiated cells have been shown to be involved in bystander signaling. Further experiments are therefore required to identify the nature of these molecules.

Taken together, these results strongly suggest that lipid rafts have opposite roles in the two HNSCC cell lines of opposite radiosensitivity. While SCC61 cells irradiation is responsible for lipid rafts production and subsequent death signaling, the endogenous presence of these platforms in SQ20B cells is probably linked to the absence of a bystander-like response and thus to resistance to ionizing radiation (Figure 7B).

It is well established that lipid rafts are enriched in PIP_2_ and PIP_3_ [53] and therefore constitute a particularly permissive region for the interaction between PDK-1 and AKT. Data from Adam et al. [37] and others [54,55] have shown that AKT protein kinases are sensitive to changes in membrane cholesterol. In the present study, we also found that treatment with MBCD induces a strong inhibition of AKT activation in SQ20B cells (Figure 6A,B), thus indicating the existence of AKT in cholesterol-enriched membranes. We hypothesize that pretreatment with MBCD causes disruption or dispersion of ceramide-enriched domains and therefore induces a change in PI3K/AKT complexes into the plasma membrane. This would mean that lipid raft integrity is critical for AKT phosphorylation in SQ20B cells. Using wortmannin as a specific inhibitor of PI3K, we demonstrated that SQ20B cells were less resistant to radiation after pretreatment with MBCD. In addition, the induction of nontargeted effects was triggered in SQ20B recipient cells preincubated with the PI3K inhibitor (Figure 6C and Figure S3B). These results suggest that inhibition of PI3K/AKT signaling may be one of the critical mechanisms involved after lipid raft disruption. The underlying mechanisms that could explain these results remain unknown and require further study.

## 4. Materials and Methods

### 4.1. Cell Lines and Cell Culture 

The human cell lines SCC61 and SQ20B were respectively derived from a tongue and larynx cancer. They were obtained from J.B. Little (Laboratory Department of Cancer Biology, Harvard School of Public Health, Boston, MA, USA) and were grown in DMEM medium supplemented with 10% heat-inactivated fetal bovine serum, 0.1 mg/mL streptomycin, 100 units/mL penicillin, and 0.4 µg/mL hydrocortisone. Cells were kept at 37 °C in a humidified atmosphere with 5% CO_2_. All culture media and supplements were purchased from Life Technologies, Inc. (Gibco BRL, Gaithersburg, MD, USA).

### 4.2. Irradiation Procedure

All experiments were performed with an X-Rad 320 irradiator (Precision X-ray Inc., North Branford, CT, USA) located at Lyon-Sud medical school (France), using 250 kV photons delivered at a dose rate of 2 Gy/min.

### 4.3. Medium Transfer Protocol, Clonogenic Survival, and Determination of Targeted and Nontargeted Effects

Cells were seeded on 25 cm^2^ flasks (200 to 2000 cells/flask) with 5 mL of medium. The following day, cells (donor cells) were irradiated at a dose varying from 0.25 to 3 Gy. For investigating the nontargeted response, the irradiated medium (conditioned medium, CM) of donor cells was collected 2 h postirradiation, centrifuged to remove any cells or debris, and transferred to a flask in which cells had been plated the day before (recipient cells 200 cells/flask). For standard clonogenic assays, donor and recipient cells were then grown for 12 days and colonies, fixed with ethanol, and stained with Giemsa. Colonies containing 50 or more cells were scored, and the surviving fraction was calculated [41]. 

To determine the contribution of targeted and nontargeted effects, we used a Bliss independence mathematical model [22,56]. It is essential to notice that donor cells are, in reality, at the same time donor and recipient cells (i.e., irradiated cells secrete substances toward neighboring irradiated cells). The Bliss model considers that donor cells could be killed either by targeted or by nontargeted effects. The following formula can be used:

Overall survival (%) = Targeted effects (%) × Nontargeted effects (%)

Overall survival was obtained considering the survival rate of donor cells and nontargeted contribution considering the survival rate of recipient cells.

### 4.4. Measurement of DNA Damage

Cells (5 × 10^5^) were seeded on a coverslip in 6-well plates. The day after, the donor cells were irradiated (1.5 or 3 Gy) and, 2 h postirradiation, the CM was transferred to recipient cells for 24 h. Both the donor (30 min postirradiation) and recipient cells were fixed in 3.7% paraformaldehyde, permeabilized in 0.5% (*v*/*v*) PBS (Phosphate Buffer Saline)/Triton X-100 for 15 min followed by incubation with 1% (*v*/*v*) PBS/BSA (Bovine Serum Albumin) for 1 h at room temperature. Cells were then incubated with mouse monoclonal anti-phospho-Histone H2A.X (Ser139, clone JBW301, Invitrogen; Saint Aubin, France) at a 1:200 dilution in 0.1% BSA/PBS overnight at 4 °C and then Alexa-488-conjugated anti-mouse secondary mAb was used at a dilution of 1:500 (Invitrogen; Saint Aubin, France) for 45 min at 37 °C. Coverslips were then mounted using Fluoromount and DAPI (Sigma-Aldrich, St Louis, MO, USA) at dilution 1:1000, and images were acquired with a 63 × NA objective with Metafer slide scanning platform (MetaSystem; Altlussheim, Germany). The quantification of γ-H2A.X foci number was obtained automatically with integrated Metafer analysis software.

The formation of micronuclei was determined in both donor and recipient cells. Thirty minutes after irradiation, in order to block cytokinesis, cytochalasin B (Sigma-Aldrich, St Louis, MO, USA) was added to the culture medium at a final concentration of 2 µg/mL and was maintained for 48 h. Cells were then harvested and centrifuged, and the pellet was treated with 125 mM KCl under constant shaking for hypotonic chock. Cells were then fixed in cold acetic acid–ethanol (1:3), stained with DAPI (1 µg/mL), and viewed using a Metafer slide scanning microscope. At least 500 cells were examined, and only micronuclei in binucleated cells were considered.

### 4.5. Immunofluorescence Detection of ASMase, Ceramide, Lipid Rafts, and p-AKT

To visualize ASMase activation and ceramide production, cells were plated in coverslips and after treatment; they were fixed with 3.7% paraformaldehyde followed by incubation with 1% (*v*/*v*) PBS/BSA for 1 h at room temperature. ASMase was then detected with a mouse anti-ASMase antibody (Santa Cruz Biotechnology, Inc., Heidelberg, Germany) at 1:100 dilution and ceramide with mouse 15B4 antibody (Alexis Biochemicals, Heidelberg, Germany) at 1:100 dilution overnight at 4 °C. Cells were then incubated with Alexa-594-conjugated anti-mouse (dilution of 1:1000) or Alexa-488-conjugated anti-mouse at a dilution of 1:500 (Invitrogen; Saint Aubin, France) for 45 min at 37 °C. Coverslips were then mounted in a Fluoromount mounting medium (Sigma-Aldrich) and analyzed using a 63 × NA objective with the Metafer slide scanning platform. Fluorescence intensity reported to the cell was automatically obtained with integrated Metafer analysis software.

For immunofluorescent co-localization of lipid rafts and AKT, cells were incubated with Ser^473^-phosphorylated AKT (S473-P AKT) primary antibody (Cell Signaling Technology, Danvers, MA, USA) diluted at 1:100 for 1 h at 37 °C followed by Alexa-555-conjugated secondary antibody (1:1000) and Alexa-488-conjugated cholera toxin B (CTxB, Molecular Probe, Illkirch, France) for 45 min at 37 °C. After three PBS washes, cells were refixed in PFA for 10 min and slides were mounted in Fluoromount mounting medium and analyzed using a Metafer microscope.

### 4.6. Lipid Raft Isolation by Successive Detergent Extraction

Lipid raft membrane fractions were performed as described in [36]. Briefly, SCC61 and SQ20B cells were harvested, rinsed, and lysed in buffer containing 25 mM 2-(*N*-morpholino)-ethanesulfonic acid, 150 mM NaCl (pH 6.5), 2% Triton X-100, 2 mM Na_3_VO_4_, and 2 mM PMSF. Lysates were incubated on ice for 30 min and then centrifuged at 14,000× *g* for 20 min at 4 °C. The supernatants (containing the Triton-soluble fraction or non-raft membrane) were removed. Insoluble pellets were resuspended in 1% Triton X-100, 10 mM Tris-Cl (pH 7.6), 500 mM NaCl, 2 mM Na_3_VO_4_, 60 mM β-octylglucoside, and 1 mm PMSF for another 30 min on ice. Triton-insoluble and raft membrane fraction supernatants were collected after 20 min of centrifugation at 14,000× *g*. All reagents were purchased from Sigma-Aldrich.

### 4.7. Western Blotting 

Cells (1 × 10^6^) were seeded in 25 cm^2^ flasks and exposed or not to radiation. Thirty minutes after irradiation, cells were harvested, rinsed, and lysed in RIPA buffer (Santa Cruz Biotechnology, Inc., Heidelberg, Germany). Cytosolic protein fractions and total lysates (30 µg of each) were separated using SDS-PAGE (12% polyacrylamide gels) and electrotransferred onto nitrocellulose membranes (Bio-Rad, Marnes-la-Coquette, France). Membranes were then incubated with anti-p-AKT (S473), AKT-pan (Cell Signaling Technology, Danvers, MA, USA), anti-flotillin-1, and anti-caveolin-1 (Santa Cruz Biotechnology, Inc., Heidelberg, Germany) primary antibodies at dilution 1:1000. Secondary antibodies were horseradish peroxidase-conjugated anti-mouse or anti-rabbit (Cell Signaling Technology), and proteins were detected using the ECL detection system Azure c300 (Azure Biosystems, Inc., Dublin, CA, USA).

### 4.8. Involvement of Lipid Rafts and PI3K/AKT Signaling in Targeted and Nontargeted Response

The role of lipid rafts’ integrity in SCC61 and SQ20B cells was investigated by preincubating SCC61 donor cells with 4 mM methyl-β-cyclodextrin (MBCD) for 30 min and SQ20B donor cells for 1 h before irradiation. Immediately after irradiation, the medium containing the lipid raft inhibitor was removed, cells were washed twice with 5 mL PBS, and fresh medium was added to cells.

The potential role of PI3K/AKT signaling was assessed by incubating SQ20B cells with 0.5 µM wortmannin for 1 h (Sigma Aldrich).

### 4.9. Proteome Profiler Human Cytokine

Human Cytokine Array Kit (Proteome Profiler Human Cytokine Array, R & D systems, Minneapolis, MN, USA) was used according to the manufacturer’s protocol to detect the expression of multiple cytokines in CM from SCC61 and SQ20B donor cells treated with irradiation alone or with MBCD combined with irradiation.

### 4.10. Cell Cycle and Apoptosis Measurement

The cell cycle distribution was determined in SQ20B and SCC61 donor cells as described previously [41] and the percentage of cells in G2/M was calculated. The effect of MBCD and wortmannin on the cell cycle was also assessed.

For apoptosis measurement, cells were plated on coverslips and allowed to attach overnight. Before irradiation, they were preincubated with MBCD, wortmannin, or not (no inhibitor). Following treatment, cells were washed with PBS and apoptosis was measured at 24 h, 48 h, and 72 h postirradiation using a TUNEL Detection Kit (Promega, Charbonnières Les Bains, France) according to the manufacturer’s instructions.

### 4.11. Statistical Analysis

GraphPad Prism v6.01 (GraphPad Software, San Diego, CA, USA) was used to performed statistical analysis. All data obtained from three independent experiments in triplicate were described using mean and standard deviation. Pairwise comparisons were performed using the nonparametric Mann–Whitney test. The significance level was set at *p* < 0.05.

## 5. Conclusions

This study showed that, in addition to targeted effects, radiation-induced nontargeted cytotoxicity can be nonnegligible and involves lipid rafts. Following irradiation, the generation of ceramide-enriched domains could, therefore, trigger apoptotic death in our radiosensitive model of HNSCC. By contrast, cholesterol depletion from the plasma membrane in radioresistant cells results in the downregulation of AKT signaling, leading to radiosensitization. These findings suggest a direct link between lipid rafts and cell survival signaling and that cholesterol-lowering drugs may be a good alternative to improve the radiotherapy effectiveness in certain cancers such as HNSCC. In this regard, data from Mantha et al. [57] and Llobet et al. [58] described promising results for statin use in HNSCC.

## Supplementary Materials

Supplementary materials can be found at https://www.mdpi.com/1422-0067/21/19/7200/s1.

## Abbreviations

ASMase	acid sphingomyelinase
CM	conditioned medium
HNSCC	head and neck squamous cell carcinoma
MBCD	methyl-β-cyclodextrin
SM	sphingomyelin
PI3K	phosphatidylinositol-3-kinase
