# Supplementary material for: Ceramide-Enriched Membrane Domains Contribute to Targeted and Nontargeted Effects of Radiation through Modulation of PI3K/AKT Signaling in HNSCC Cells
Source: Int J Mol Sci. 2020 Sep 29;21(19):7200. doi: 10.3390/ijms21197200 (PMC7582380; doi:10.3390/ijms21197200)
Supplement: Supplementary file 1 [file ijms-21-07200-s001.pdf]

Table S1. Quantification of ASMase and ceramide levels in SCC61 and SQ20B cells: ASMase externalization and ceramide generation were measured using immunofluorescence and fluorescence quantified with integrated Metafer microscope software (MetaSystem; Altlussheim, Germany). The results are the mean ± SD of three experiments performed in triplicate. *$p < 0.05$, **$p < 0.01$, and ***$p < 0.001$ compared with untreated cells.

	ASMase level (mean intensity/cell)		Ceramide level (mean intensity/cell)	
	SCC61 cells	SQ20B cells	SCC61 cells	SQ20B cells
**Untreated cells**	0.12 ± 0.04	0.58 ± 0.17	0.08 ± 0.02	0.26 ± 0.06
**10 mM H2O2**	0.54 ± 0.12 ***	0.62 ± 0.08	0.19 ± 0.06 **	0.19 ± 0.04
**Donor cells**	0.31 ± 0.08 **	0.66 ± 0.10	0.25 ± 0.07 **	0.21 ± 0.07
**Recipient cells**	0.21 ± 0.04 *	0.47 ± 0.09	0.20 ± 0.09 *	0.16 ± 0.06

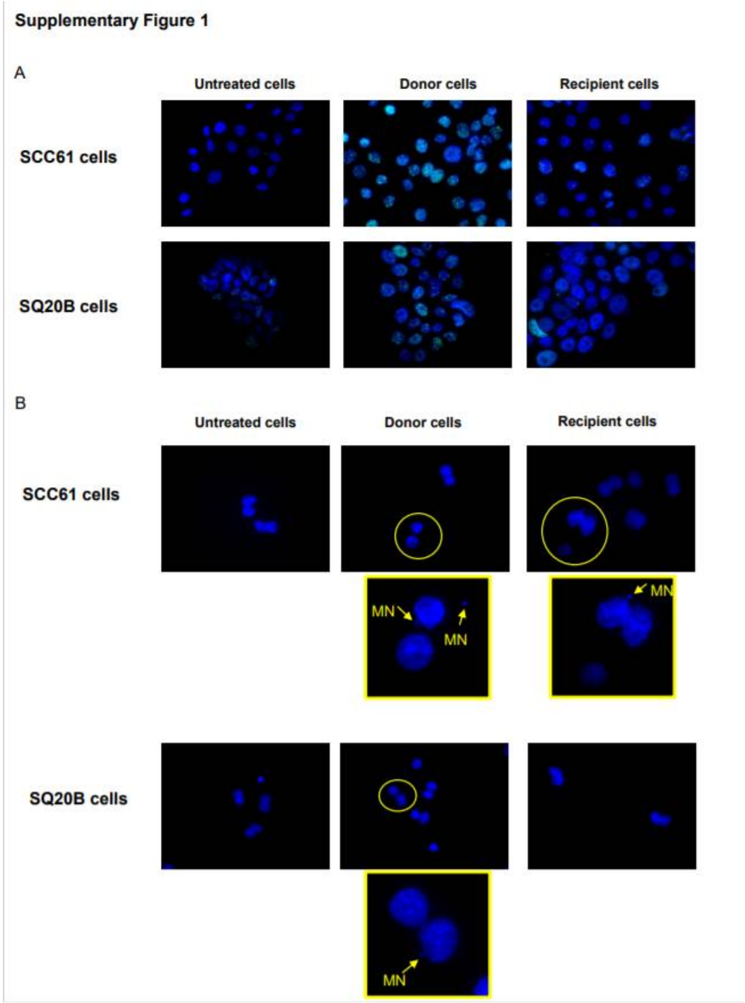

Figure S1. DNA damage measured in donor and recipient cells following exposure to X-rays (A) The average number of ɣ-H2A.X foci per cell was quantified after immunofluorescence labeling in irradiated SCC61 and

SQ20B cells and in their corresponding recipient cells. (B) The yield of micronuclei per binucleated cell was also determined in SCC61 and SQ20B donor cells and in corresponding recipient cells.

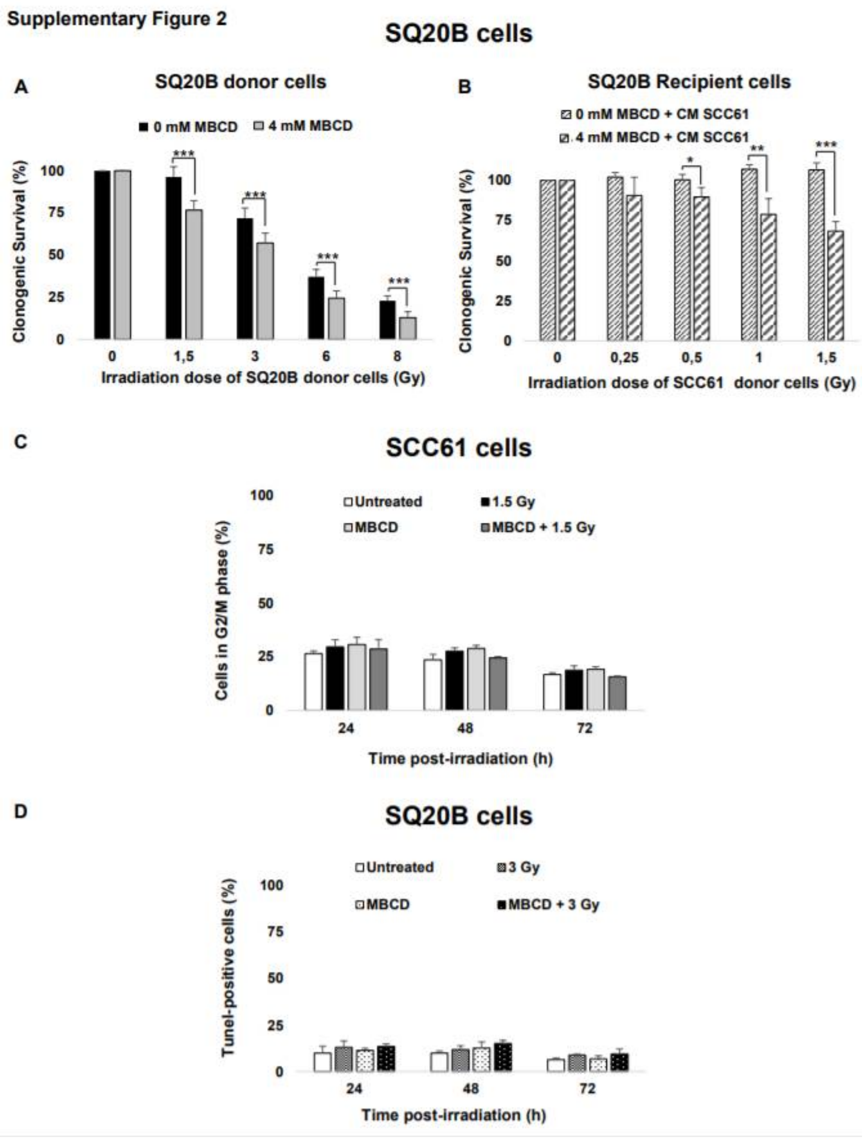

Figure S2. Clonogenic survival, cell cycle analysis, and apoptosis measurement: (A) Clonogenic survival was determined in SQ20B donor cells pretreated with or without MBCD before irradiation (high doses). (B) Recipient SQ20B cells were also treated with or without MBCD before transfer of SCC61 CM, and clonogenic survival was assessed. (C) The percentage of SCC61 cells in the G2/M cell cycle following irradiation or MBCD + irradiation was determined. (D) Apoptosis was measured using a TUNEL assay in SQ20B donor cells preincubated or not with 4 mM MBCD for 60 min before irradiation. The results are the mean ± SD of three experiments performed in triplicate. *$p < 0.05$, **$p < 0.01$, and ***$p < 0.001$ compared with cells treated only with radiation.

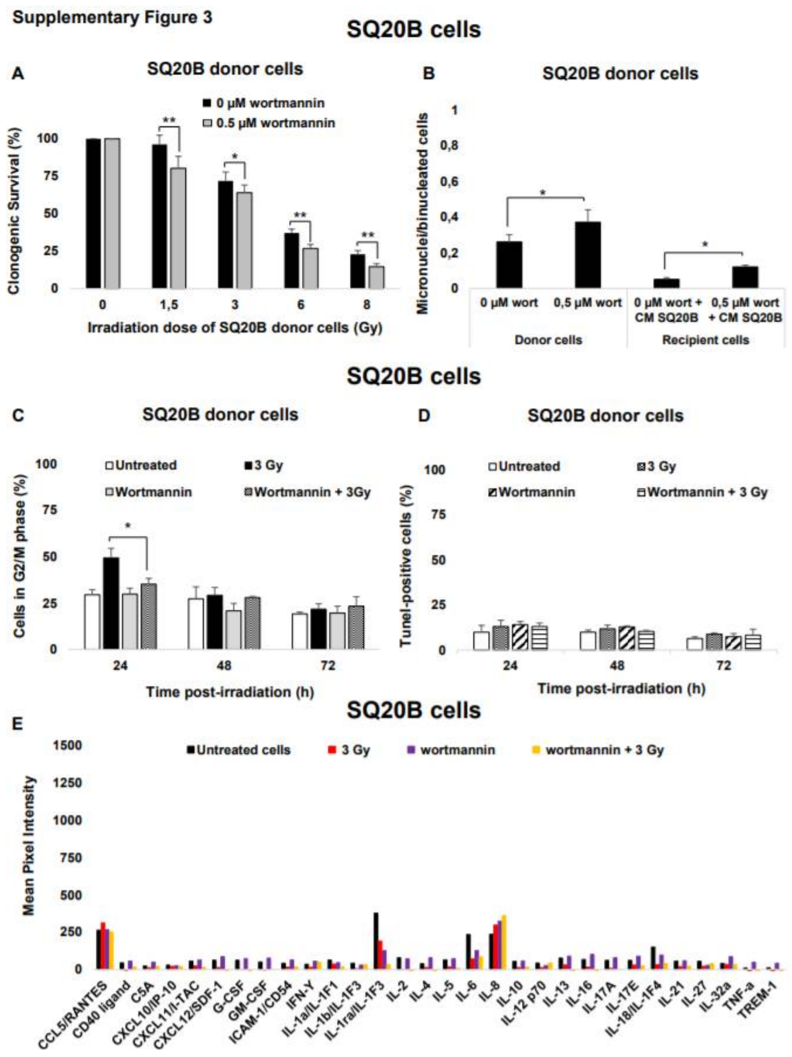

Figure S3. Clonogenic survival, DNA damage, cell cycle analysis, and apoptosis measurement: (A) clonogenic survival of SQ20B donor cells pretreated with or without wortmannin (PI3K inhibitor) before irradiation (high doses), (B) quantification the number of micronuclei per cell in SQ20B donor and recipient cells in the presence or absence of a PI3K inhibitor, (C) the percentage of SQ20B donor cells in the G2/M phase following irradiation or MBCD + irradiation, and (D) apoptotic death measured in SQ20B donor cells pretreated with or without a PI3K inhibitor using a TUNEL assay. The results are the mean ± SD of three experiments performed in triplicate. **p* < 0.05, ***p* < 0.01, and ****p* < 0.001 compared with cells treated only with radiation. (E) The expression of cytokines secreted in the CM from untreated, irradiated (3 Gy), and wortmannin + irradiated SQ20B cells was evaluated using the Human Cytokine Array (Proteome Profiler Array; R&D Systems, Minneapolis, MN). For each cytokine, ImageJ software was used to determine pixel intensity.
